# Prevention of neuroinfectious-diseases: high impact of vaccination programs – but the potential is not maxed out yet

**DOI:** 10.1186/s42466-026-00489-1

**Published:** 2026-05-11

**Authors:** Matthias Klein, Maximilian Schöls, Hayrettin Tumani, Uta Meyding-Lamade, Julia Decker, Matthias Maschke, Judith Wagner

**Affiliations:** 1 Department of Neurology, Klinikum Grosshadern, Ludwig-Maximilians University, Marchioninistr. 15, Munich, 81377 Germany; 2 Emergency Department, Klinikum Grosshadern, Ludwig-Maximilians University, Munich, Germany; 3 Department of Neurology, Charite, Berlin, Germany; 4 Department of Neurology, University of Ulm, Ulm, Germany; 5 Department of Neurology, Krankenhaus Nordwest, Frankfurt, Germany; 6 Department of Neurology, Immanuel Klinik, Universitätsklinikum Rüdersdorf der MHB (Medizinische Hochschule Brandenburg), Rüdersdorf, Germany; 7 Department of Neurology, Brüderkrankenhaus, Trier, Germany; 8 Department of Neurology and Neurorehabilitation, Evangelisches Klinikum, Gelsenkirchen, Germany

**Keywords:** Vaccination, CNS infection, Bacterial meningitis, Tick-borne encephalitis, Neuroborreliosis, Varicella zoster virus, Measles

## Abstract

**Background:**

The greatest impact on the burden of CNS infections resulted from preventive measures, mainly vaccination programs, that have decreased the incidence of many CNS infections significantly. Here, we highlight the main cornerstones of vaccination programs on bacterial and viral CNS infections and point at future chances of upcoming vaccines.

**Main body:**

Vaccination programs have significantly decreased the number of cases due to Haemophilus influenzae type B and Neisseria meningitidis. For pneumococcal meningitis, new vaccines that prevent neuroinvasive serotypes have the potential to decrease numbers in the future. Whereas a vaccine is available for tick-borne encephalitis, prevention of neuroborreliosis is limited to contact precautions. However, vaccination rates against tick-borne encephalitis remain too low in many countries. Another neurotropic virus that can be prevented effectively by vaccination is varicella zoster virus (VZV). Interestingly, vaccination against VZV also seems to reduce the risk of dementia as recently shown. While vaccination is important in general, patients with immunosuppression benefit most from vaccinations. Finally, recent measles outbreaks impressively underline the efficacy of vaccination programs and demonstrate what can happens if vaccination rates decrease.

**Conclusion:**

Vaccination programs have shown to be effective in reducing the burden of CNS infections. As vaccination rates are generally still too low and as new vaccines are coming up, it is obvious that the potential of vaccines to prevent CNS infections is not maxed out yet.

## Introduction

Neuroinfectious diseases are a challenge to diagnose, still have a high mortality and survivors often suffer from neurological sequelae. Rapid diagnostic workup and immediate initiation of the anti-infective therapy (often initially combined with acyclovir) in the emergency department is key to improve outcome [1, 2]. Similar to other infectious diseases, prevention—mainly through consequent vaccine programs—had a strong impact on worldwide deaths secondary to neuroinfectious diseases. In the following review, we focus on the most important milestones in the prevention and highlight crucial recommendations on the prevention of infections of the nervous system.

## Bacterial meningitis

### Burden of the disease

Bacterial meningitis remains a disease with a fatality rate of 10–15% in adults, varying by pathogen [1, 3]. Fortunately, vaccination programs have made bacterial meningitis almost a rare disease with an incidence of approximately 1.6 episodes per 100,000 population per year in Central European countries nowadays. In comparison, the incidence was as high as 6.4 per 100,000 population per year in the early nineties [4, 5].

### Impact of vaccination programs – haemophilus influenzae

The success of vaccination programs with respect to bacterial meningitis started with the introduction of the vaccine against *Haemophilus influenzae* Type B (HiB) in the early 1990s. The vaccine was the first conjugate vaccine on the market and wherever vaccination programs were introduced, rates of HiB meningitis dropped dramatically within a very short time. For example, in the Netherlands, the incidence of HiB meningitis declined from 1.44/100000 per year in the 1991–1993 to 0.04/100000 per year in 2000–2002 [4]. The vaccine was introduced for children (the age group with the highest incidence of HiB meningitis) leading to a decline from 22.94 to 0.46/100000 per year. Interestingly, vaccination of children also led to a decline of HiB meningitis cases in nonvaccinated age groups at a similar rate (IRR, 0.02 [95% CI, 0.02–0.04), an effect that can be explained by herd immunity.

### Impact of vaccination programs – neisseria meningitidis

The second milestone in preventing bacterial meningitis resulted from the introduction of vaccines against *Neisseria meningitidis*. The first vaccine became available in the late 1990s. In *Neisseria meningitidis*, however, the situation is more complex as meningococcal meningitis cases cannot be attributed to one serotype only. Serotype distribution differs worldwide: in Europe, the Americas and Australia, serotype B, C, W, and Y account for most of the cases. In the African so-called meningitis belt, serotypes A, C, and W dominate [6]. First available effective vaccines were directed against serotype A, C, W, and Y and later also serotype B and their directed use according to local predominance of certain serotypes led to a decrease of the incidence. E.g., the introduction of the serotype C vaccine in the Netherlands after an outbreak led to a dramatic decline of MenC meningitis cases from 0.62/100000 to 0.01/100000 per year [4]. In Germany, the number of cases dropped by 50% (Table 1). The introduction of vaccines, natural variations after periods of a predominant genotype in one area, and spread by travel and major events like the Hajj pilgrimage lead to a change of serotype distribution worldwide [7, 8]. In consequence, vaccination programs need to be carefully re-evaluated on a regular basis. A recent example of the need of worldwide surveillance programs is the current development of serotype distribution for invasive meningococcal disease: During the COVID-19 pandemic, a significant decrease of invasive meningococcal diseases was observed, mainly due to contact precautions and social distancing. Not surprisingly, numbers increased again after the pandemics. As an example for Central European trends, in Germany, the total number of registered invasive meningococcal disease cases was down to 68 cases in 2021 but increased back to 324 cases in 2024 [9, 10]. Interestingly, serotype Y is now found to be the main causative strain which had not played a significant role in Germany in the years before the pandemic. As a consequence, vaccination against serotype Y has now been recommended. Data from the US also illustrate impressively how important vaccination surveillance is: although vaccination rates in adolescents reach 89% coverage for Serotypes A, C, W and Y, only 29% have received a vaccine against serotype B (although serotype B is responsible for 26–38% of cases) [11]. Hopefully, the introduction of a combination vaccine against serotype A, B, C, W, and Y that was released in 2025, will close this vaccination gap. In addition, strict contact precautions for medical staff when treating patients with invasive meningococcal disease and chemoprophylaxis for close contacts are effective to prevent spread of the disease.

**Table 1:** Impact of vaccination programs on the incidence of acute bacterial meningitis

Disease	Region	Year of implementation of Vaccination Program	Incidence prior to vaccination (per 100,000 per year)	Incidence after implementation of vaccination	Special Aspects
*Haemophilus influenzae*—meningitis	Netherlands [4]	1993	1.44 (in 1991–1993)	0.04 (in 2000–2002)	Coverage for Serotype B; Vaccine introduced for children
*Neisseria meningitidis* -meningitis and sepsis	Netherlands [4]	2002 (MenB)	0.62 (Serotype C, in 200–2002)	0.01 (Serotype C, 2009–2011)	Prior to vaccination, Serotype C caused 41% of all invasive meningococcal diseases
*Neisseria meningitidis* -meningitis and sepsis	Germany [10, 79]	2006 (MenB	0,2–0.26 (Serotype C, in 2001–2004)	0.01 (Serotype C, in 2022–24)	Dominant serotype in Germany is serotype B, vaccine against serotype B recommender since 2024
*Streptococcus pneumonia* – meningitis	Netherlands [4]	2006 (PCV 7)	0.76 (Serotypes included in PCV7 vaccine, in 2004–2006)	0.03 (Serotypes included in PCV7 vaccine, in 2018–2019	Further vaccines covering additional serotypes (such as PCV10, PCV15, PPSV23) were introduced in the following years
*Streptococcus pneumonia*—meningitis	United States [12]	2000 (PCV7) 2010 (PCV13), 2021 (PCV15 und PCV20), 2024 (PCV 21)	0.8 (all serotypes, in 2008–2009)	0.6 (all serotypes, in 2022–2023)	Reduction can be attributed to a reduction of serotypes covered by PCV13
*Tick borne encephalitis*	Austria [31, 80]	1981	-	Non-vaccinated: 6.5 (in 2020–2024) Vaccinated: 0.09 (in 2020–2024)	Austria achieved a high TBE vaccination coverage (85%). Differences in the incidence rates among vaccinated and non-vaccinated individuals impressively demonstrate the success of the vaccine

### Impact of vaccination programs – Streptococcus pneumoniae

Currently, the main pathogen causing bacterial meningitis in developed countries is *Streptococcus pneumoniae* [12]. Vaccination programs have started with a polysaccharide vaccine against 14 serotypes, followed by a 23-valent vaccine a few years later in the late ´70s and early ´80s., covering 90% of invasive pneumococcal strains. These polysaccharide vaccines, however, failed to produce sufficient protection especially in young children and in the elderly, the most vulnerable population to invasive pneumococcal disease. The next milestone was achieved by the introduction of the conjugate vaccines PCV7, PCV-10, and PCV13. Although the effect on invasive pneumococcal infections such as pneumococcal pneumonia was strong, only few S. pneumoniae serotypes that cause pneumococcal meningitis were included and the emergence of strains not included in the vaccine (serotype replacement) somewhat cancelled out the vaccination effect [13, 14]. Thus, it is not surprising that during the last years, the rates of pneumococcal meningitis remained stable in most countries, apart from a temporary drop of pneumococcal meningitis cases during the COVID-19 pandemic [12, 15]. Hopefully, the introduction of new vaccines such as PCV 15 (approved in Europe and the US in 2021), PCV 20 (approved in Europe in 2022 and in the US in 2023), and PCV21 (approved in the US in 2024 and in Europe in 2025) covering further strains (including especially strains that are known to have been among the main meningitis-causing cases in the last decades) will lead to a significant decline in adult meningitis cases.

Another interesting question is, whether pneumocccal vaccination can reduce the occurrence of meningitis in certain risk groups. In a study on 35,434 patients who received a cochlear implant, vaccination against pneumococci was associated with a significantly lower rate of pneumococcal meningitis [16]. Thus, vaccination in specific risk groups is recommended in most countries.

### Special aspects and challenges

Besides *S. pneumoniae* and *N. meningitidis*, *Listeria (L.) monocytogenes* remains the third main causative pathogen for community-acquired bacterial meningitis [5]. Infections with listeria are mainly found in certain risk groups including breast-fed infants, immunocompromised patients and the elderly. Unfortunately, vaccines against *L. monocytogenes* are currently not available. Being a mainly food-borne pathogen, prophylaxis against *L. monocytogenes* can be achieved only by avoiding products with a high risk of contamination with listeria, such as raw milk products (such as raw milk itself or cheese from raw milk) or fresh meat or seafood [17]. Also, outbreaks of infections by *L. monocytogenes* that are mediated through commercially available food in supermarkets occur on a regular basis [18]. Public health surveillance programs that are able to link cases that occur geographically far apart after genotyping isolated strains from infected patients have successfully led to a rapid identification of contaminated food products, allowing to withdraw them from supermarket shelves [19, 20].

The leading pathogens in neonates are Group B Streptococcus (GBS) and Escherichia coli. Routine intrapartum antibiotic prophylaxis—specifically intravenous penicillin G administered during labor to GBS-colonized mothers—has significantly reduced early-onset GBS disease but does not prevent late-onset cases [13, 21]. Efforts to develop maternal GBS vaccines are ongoing [22].

Taken together, the incidence of acute bacterial meningitis was significantly decreased by the introduction of effective vaccination programs, especially for *H. influenzae* and *N. meningitidis*. In terms of *S. pneumoniae*, new vaccines have the potential to even further decrease the number of patients, hopefully making it a rare disease in the near future. Progress will depend on closing existing vaccination gaps, using pneumococcal vaccines with broader serotype coverage, maintaining robust surveillance to guide program updates, and preventing listeriosis through food hygiene measures.

## Tick borne encephalitis

### Burden of the disease

Tick-borne encephalitis (TBE) is a potentially serious disease caused by infection with an RNA virus from the flavivirus family. The most important vectors are ticks—in Central Europe primarily Ixodes ricinus. Small rodents are the main reservoir host. Cases of transmission through infected raw milk are much rarer [23]. Symptomatic infections (5–30%) can involve any part of the nervous system. Particularly severe courses are seen in the context of TBE myeloradiculitis. The mortality rate in adults in Central Europe is approximately 1% [24]. According to the Robert Koch Institute (RKI), the TBE incidence in Germany has tripled between 2002 and 2022 compared to the previous 20-year period [9, 25], a trend that has also been observed in other central and especially Eastern European countries [26–29].

### Impact of vaccination programs

A vaccine against TBE is available since the 1970s. Its introduction caused a sharp decline of TBE cases in countries that achieve a high coverage [30]. The highest vaccination coverage has been achieved in Austria where approximately 85% of patients are vaccinated. Incidence in vaccinated patients is as low as 0.09/1000000 per year compared to 6.5/10000 per year in unvaccinated individuals [31]. In contrary, rates in Germany have been increasing despite the vaccine being available as vaccination rates remain too low.

When visiting or living in an endemic area, TBE vaccination is strongly recommended. Two vaccines (TBE-IMMUN®, ENCEPUR®) are available in Europe. Three vaccinations are required for basic immunization. The first booster vaccination is recommended for both substances after three years, all further vaccinations after 5 years (or after 3 years depending on the age). Unfortunately, vaccination coverage remains low although patients are aware of the availability of the vaccine. A study on patients with tick borne encephalitis revealed that unawareness of the real infection risk and concerns about adverse reactions of the vaccine keep people from being vaccinated [32]. For example, full vaccination is achieved in only 35% of individuals at the age 50 to 60 in southern Germany, where TBE is endemic [33]. This is regrettable as all vaccines are generally well-tolerated [34].

### Special aspects and challenges

Meanwhile however, the geographical spread and overall incidence in Europe is increasing, primarily due to climatic changes. This increase in TBE incidence is due to changes in human leisure behavior and the increased spread and activity of the vector Ixodes ricinus. This requires a minimum temperature of approx. 5°C and a relative humidity of at least 80%. Increasingly mild winters and humid summers therefore promote horizontal and vertical geographical spread and cause an extension of the activity period.

As no specific therapy is available for TBE, prophylaxis is essential, i.e. by wearing clothing that covers the skin and the use of repellents. After spending time outdoors, the skin should be searched carefully and ticks should be removed by pulling slowly with fine-tipped tweezers. Consumption of unpasteurized dairy products is to be avoided.

TBE may serve as a model disease for other Flavivirus infections. These – such as West Nile encephalitis – rank among the most common viral CNS infections worldwide and are emerging infections in South and Central Europe (P. [35, 36]). Surveillance and other public health infrastructure developed for TBE may be applied to these pathogens as well.

## Lyme neuroborreliosis

### Burden of the disease

Lyme borreliosis (LB) including neuroborreliosis is the most frequent tick-borne disease in Europe and the United States.It is caused by an infection with the bacterium *Borrelia burgdorferi* sensu lato through the bite of an infected tick. Erythema migrans is the most common early sign of infection appearing several days or weeks after infection and might be accompanied by unspecific symptoms such as fever, fatigue, headaches and arthralgias. The course of untreated patients can be complicated by Lyme neuroborreliosis in about 3% with intense radicular pain, paresis and sensory symptoms as well as severe headache (Bannwarth’s syndrome) or Lyme carditis in < 1% [37].

According to recent surveillance data from 29 European countries, the incidence exceeds 100 cases per 100.000 person-years [38]. In parallel with the rising incidence of tick-borne encephalitis, the incidence of Lyme borreliosis increased by approximately 36% within the last two years of surveillance, which is mostly due to climate change with warmer temperatures and recent increase in outdoor activities [39]. In Germany, the average annual LB incidence was 37.2/100.000 person-years and varied by spatial level, ranging from 22.9 to 64.6/100.000 person-years according to an analysis between 2016–2020 [40].

### Impact of vaccination programs

In 1998, a vaccine against borrelia was licensed in the United States (LYMErix®). Because of reported side effects in some patients (arthritis) and low demand, the vaccine was taken off the marked. However, a direct relationship between the vaccine and new onset arthritis was not found. Currently, there is no vaccine available to prevent Lyme borreliosis in humans.

Recent studies using mRNA vaccines that target the outer surface protein A (OspA) are promising. Data of vaccine studies in mice demonstrates that an mRNA-OspA vaccine can be effective against LB infection [41]. In humans, an ongoing Phase 3, multicenter, placebo-controlled, randomized, observer-blinded trial evaluates the efficacy and safety of a 6-Valent OspA-based Lyme disease vaccine in healthy participants ≥ 5 years of age (NCT05477524, registration date July 25th, 2022). The concept of the vaccine is that antibodies against OspA are taken up by the tick with blood during the act of suction. These antibodies are meant to bind against borrelia inside the tick´s gut and, in consequence, prevent borrelia from entering the human victim. First results are promising [42], [43, 44].

### Special aspects and challenges

Similar to tick borne encephalitis, prophylaxis begins with personal protection such as wearing of permethrin treated clothing/uniforms, application of spray repellents to skin or clothing and preventive behaviors such as tick-habitat avoidance, checking for ticks after outdoor activities and showering/bathing [45]. An open-label randomized controlled trial tested the use of post exposure administration of antibiotics after tick bite. In this trial, a single dose of 200 mg doxycycline within 72 h after removing an attached tick from the skin was administered, and compared infection rates to no treatment [46]. Results showed a relative risk reduction of 67% in treated patients with a number needed to treat of 51. Similar results were found in earlier trials [47]. However, number needed to treat is high and doxycycline might have a negative effect on gut flora. International guidelines differ. Whereas the Infectious Disease Sociaty of America (IDSA) recommends a single dose of doxycycline within 72h after tick bite in regions where borrelia are endemic, German guidelines do not recommend post exposure prophylaxis [48], [49].

Other preventive interventions such as landscape management, host animal parasitism and movement as well chemical/natural/botanical applications to reduce the number of ticks are theoretically appropriate interventions. Factors like social acceptability and resistance, environmental impact, cost, and feasibility will, however, interfere with the implementation of those interventions [45].

## Varicella zoster virus

### Burden of the disease

The varicella-zoster virus (VZV) is a double-stranded DNA virus belonging to the Alphaherpesviridae subfamily and is classified as *human herpesvirus 3* (HHV-3). It is closely related to herpes simplex viruses type 1 and 2 (HSV-1 and HSV-2). During primary infection, VZV causes chickenpox (varicella) and subsequently establishes lifelong latency in sensory ganglia. Prior to the introduction of the attenuated live vaccine (1995 in the US, 2004 in Germany), most children would acquire varicella before the age of 10. Although generally self-limiting, varicella can result in serious complications, including central nervous system infections, particularly in immunocompromised children. Reactivation of latent VZV leads to herpes zoster (shingles), characterized by painful, dermatomal vesicular eruptions [50]. Incidence is age dependent and ranges from 5.49 to 9.85/1000 in Europe and the US in adults ≥ 50 years of age [51]. About 8 – 21% of patients develop postherpetic neuralgia (PHN), a debilitating condition marked by persistent neuropathic pain that may last for years [52]. In addition to cutaneous manifestation and PHN, VZV reactivation can cause (meningo-)encephalitis of varying severity or other serious neurological disorders [53]. A rare but severe complication is VZV-associated cerebral vasculopathy which can ultimately cause ischemic stroke.

### Impact of vaccination programs

Where adopted, the introduction of vaccination programs against VZV in children (attenuated live vaccine with the vOka strain) led to a marked reduction in incidence of varicella and varicella-related complications [54–57]. The vaccine virus persists in ganglia and can occasionally reactivate as herpes zoster, though this is far less common than after wild-type VZV infection [58].

A live attenuated vaccine against herpes zoster became available in 2006, but its limited efficacy prevented its recommendation and widespread adoption in many countries [59]. A major advance was the approval of the recombinant subunit vaccine (Shingrix®) in 2016, which has shown superior and long-lasting protection [60]. In Germany, the Standing Committee on Vaccination (STIKO) has recommended Shingrix since 2018 as a standard vaccination for adults aged 60 years and older, and as an indication-based vaccination for immunocompromised individuals from age 50. However, acceptance among adults over 60 years remains suboptimal in most countries with low to very low vaccination rates [61].

Respectively, in many countries, the incidence of VZV-related hospitalizations are still not decreasing in several countries. This is, among others, due to an aging population and an increase in immunocompromised patients who are at special risk for VZV reactivation [62].

### Special aspects and challenges

Recently, attention has turned to the potential role of VZV in neurodegeneration. Several high-impact studies published in 2025 have reported a reduced risk for dementia after zoster vaccination [63–66]. The proposed mechanism is the prevention of subclinical viral reactivations, which may otherwise trigger neuroinflammation. Alternative hypotheses include reduced viral reactivation or yet unidentified immunological effects. Interestingly, preliminary data suggest that women benefit more than men.

Taken together, VZV is not only a classic childhood pathogen but also a major contributor to morbidity in older adults with substantial impact on quality of life. (Meningo-)Encephalitis and cerebral vasculopathy are rare but significant complications of VZV infection and reactivation. The introduction of the attenuated live vaccine against VZV led to a pronounced reduction in varicella-associated morbidity and mortality. The recombinant subunit vaccine effectively prevents herpes zoster and its complications, yet vaccination coverage remains unsatisfactory, and warrants intensified public health efforts. If the emerging evidence for dementia prevention is confirmed, VZV vaccination could reach far-reaching impact beyond infectious disease control.

## Measles

### Burden of the disease

Measles is a highly contagious viral infection caused by a pathogenic enveloped RNA virus belonging to the genus Morbillivirus (family Paramyxoviruses). It is transmitted through inhalation of infectious droplets or airborne particles as well as by contact with infectious secretions from the nose or throat. In addition to acute symptoms and a characteristic patchy, nodular measles rash on the skin, measles carries a significant risk of serious complications, including neurological manifestations. Consistent vaccination adherence is therefore crucial, not only to prevent the disease itself, but also to minimize the risk of irreversible neurological sequelae.

Measles can cause serious neurological disorders: Primary measles encephalitis (PME) and acute post-infectious measles encephalitis (APME) are particularly serious complications. PME occurs in approximately 1 in 1,000 cases. It occurs approximately 4–7 days after the onset of the rash, with headache, fever and impaired consciousness, even coma. Measles inclusion body encephalitis (MIBE) occurs predominantly in immunocompromised patients approximately 5 weeks to 6 months after infection and is characterized by lethargy, fever, weakness, dysarthria, epileptic seizures, and progressive impaired consciousness. The mortality rate is 30%.

Subacute sclerosing panencephalitis (SSPE) is a very rare late complication that manifests an average of 6–8 years after infection. According to the literature, there are an average of 4–11 SSPE cases per 100,000 cases of measles. Children are at significantly higher risk. The risk of developing SSPE has been estimated at 30–60 per 100,000 cases of measles for children who contract measles before the age of 5, and as high as 170 per 100,000 cases for children who contract measles in their first year of life. Beginning with psychological and intellectual changes, the disease progresses to a course with neurological disorders and deficits, leading to the loss of cerebral functions. In approximately 10–20% of those affected, it is fatal; in approximately 20–30%, permanent damage to the central nervous system must be expected. In recent years, there has been an increase in its incidence as a consequence of a reduction in vaccination adherence.

### Impact of vaccination programs

For measles, highly effective vaccines were developed and released in the 1960s. Before the first measles vaccine was approved in 1963 in the US, there were an estimated 3 to 4 million cases of measles annually in the United States, resulting in 400 to 500 deaths and 48,000 hospitalizations. The Centers for Disease Control and Prevention declared measles eliminated in the United States in 2000.

### Special aspects and challenges

Despite the availability of a highly effective vaccine, a decline in vaccination coverage is leading to renewed outbreaks of measles – with serious consequences. According to WHO and UNICEF, vaccination coverage in Europe in 2024 remained below pre-pandemic levels, falling from 92 to 91% for the second dose of MMR in particular. However, at least 95% coverage is required for effective herd immunity. The WHO decline in vaccination rates in Europe and the US increases the risk of new measles outbreaks.

The recent measles outbreaks in the USA have also been attributed to declining vaccination rates. As of July 23, 2025, a total of 1,319 cases were reported, most of them (92%) in unvaccinated individuals or those with unknown vaccination status in communities with low vaccination coverage of 70% or 80% only [67]. In the US, a model study published in JAMA (April 2025) shows alarming projections: a 10% decline in vaccination rates could lead to over 11 million measles cases within 25 years, including thousands of neurological complications and hundreds of deaths.

In the WHO European Region, approximately 89,000 people suffered from measles in 2018 and in 2019; the number of measles cases increased by about 20% to about 106,000, 64 people died. In 2022, after the pandemic when numbers had declined, the number of measles cases in Europe rose significantly again from 937 cases in 2022 to 58,000 in 2023, particularly among children under 5 years of age – a warning signal for renewed awareness and intervention.

In summary, many steps remain to be taken to eradicate measles. Given the limited available treatments, compliance with measles vaccination recommendations is the only effective strategy for preventing life-threatening neurological diseases such as MIBE and SSPE. The COVID-19 pandemic has resulted in dangerous immunity gaps due to suspended awareness campaigns and delayed vaccination activities. Therefore, there are serious medical concerns about the risk of new measles outbreaks in the coming years. The fatal course of neurological manifestations underscores the importance of vaccination programs and requires urgent action. An intensified vaccination campaign combined with evidence-based education is essential to ensure both individual health and herd immunity in the long term.

## Special aspects in immunocompromised patients

### Burden of the disease

Neuro-infectious diseases remain a significant cause of morbidity and mortality, particularly among immunocompromised individuals [68]. E.g., the risk for patients with defects in the complement system (C5-C8) to suffer from invasive meningococcal disease is increased up to factor 7000 and patients under therapy with the complement inhibitor eculizumab encounter an increased risk of factor 1000–2000 [69]. Overall, prevention in vulnerable immunocompromised patients is key [70, 71].

This is complicated as patients with hematologic malignancies, those on B-cell depleting agents or other agents against demyelinating disease, and transplant recipients have the poorest seroconversion rates after vaccination. However, a missing seroconversion after vaccination does not necessarily reflect a completely missing vaccination response as a T-cell response might also provide protection after vaccination. In contrary to patients on immunocompromising drugs, those with solid tumors or stable HIV (normal CD4 counts) demonstrate acceptable responses [70].

### Impact of vaccination programs

Apart from the vaccination indications for immunocompromised patients that are discussed in the previous paragraphs, annual inactivated influenza vaccination and pneumococcal vaccination are recommended for all immunocompromised patients, as these pathogens are common causes of neuro-infectious complications (e.g., meningitis, encephalitis). Vaccine effectiveness is often reduced in immunocompromised patients, but strategies such as high-dose or adjuvanted vaccines, booster doses, and timing vaccination during periods of minimal immunosuppression may improve effectiveness [71–73]. Benefits of vaccination clearly appear to outweigh the risks. As a consequence, the American Academy of Neurology (AAN) and the European Committee for Treatment and Research in Multiple Sclerosis (ECTRIMS) / European Acadamy of Neurology (EAN) consensus recommend that patients with multiple sclerosis (MS) receive all indicated vaccinations. These principles extend to other neuroimmunological and neuromuscular disorders [71].

### Special aspects and challenges

Optimal timing of vaccination is crucial, and vaccines should generally be administered before initiation of immunosuppressive therapy when feasible, or during periods of lowest immunosuppression (Table 2). However, if an immunosuppressive therapy is urgently indicated, vaccination should not delay treatment. One example is a highly active NMOSD that demands therapy with a complement inhibitor. In such cases, treatment should be started but as complement inhibitors increase the risk for meningococcal infection, a two weeks daily antibiotic chemoprophylaxis needs to be ensured until the vaccination provides protection against meningococci [74].

**Table 2:** Recommendations for vaccination in immunocompromised patients

Key recommendations for vaccination in immunocompromised patients
Complete vaccinations before initiating immunosuppressive therapy whenever possible
Avoid live vaccines during active immunosuppression
Account for reduced responses with B-cell–depleting therapies; vaccinate ≥ 6 months after the last infusion if feasible
Restart immunization schedules after hematopoietic stem cell transplantation from approximately 3–6 months post-procedure
In asplenia or complement inhibition, ensure coverage with PCV, MenACWY, MenB, and Hib vaccines
Vaccinate close household contacts to provide indirect protection

Unfortunately, unjustified safety concerns keep patients from getting vaccinated: Inactivated vaccines are generally safe and recommended and only live-attenuated vaccines are contraindicated in patients with significant immunosuppression due to the risk of vaccine-associated disease [73, 75].

In many countries, vaccination recommendations for immunocompromised patients exceed the general vaccination recommendations. For example, usage of PCV20 for pneumococcal vaccination is recommended in Germany for immunocompromised children and adults. Furthermore, VZV vaccination using Shingrix is recommended in all immunocompromised persons at the age of 18 or older. Caution is required in the use of live vaccines in immunocompromised patients.

In summary, vaccination is central to the prevention of neuro-infectious diseases in immunocompromised patients. Strategies should be individualized according to immune status, treatment timing, and pathogen exposure, while ongoing research continues to refine evidence-based guidance and address current knowledge gaps.

## Travel-associated CNS infections

### Burden of the disease

Travel-associated infections are illnesses acquired while traveling. The spectrum of what diseases cause a so-called “travel-associated” infection is highly dependent on the place of primary residency. From a European perspective, following traveler’s diarrhea, dengue, malaria, and chikungunya occur often simply because these infections have a high prevalence in certain regions of the world. For example, 50 to 100 million patients suffer from dengue virus infections worldwide, mainly in Southeast Asia, Southern America and Africa [76]. the most common travel-associated infections have short incubation periods and, therefore, the majority of travelers will seek medical attention within one month of returning home. Dengue and other arboviral infections, as well as influenza usually show shorter incubation periods of about 2 weeks and less. Awareness is key in diagnosing travel-associated infections.

There are only few vaccines available for travel-associated diseases. Here, we will briefly focus on vaccines against Chikungunya virus, Dengue fever, tuberculosis, and rabies.

### Impact of vaccination programs – chikungunya virus

There are two vaccines available, a live attenuated vaccine (called IXCHIQ) and a virus-like particle vaccine (called VIMKUNYA). Travelers who are at higher risk of exposure to chikungunya virus should consider getting vaccinated before their trip. As several countries report serious adverse events of the vaccine, including hospitalizations for cardiac and neurologic events, in August 2025 FDA has suspended IXCHINQ whereas in Europe, it is still available. Vaccination against Chikungunya virus is currently recommended in most countries for travelers with an increased risk of exposure, e.g. when travelling to a country with a current Chikungunya outbreak or if a prolonged stay is planned in an endemic region and a high risk for severe disease exists (elderly patients or in the case of severe medical conditions).

### Impact of vaccination programs – dengue virus

The virus is transmitted by mosquitoes and is endemic in most tropical and sub-tropical countries. Overall, there is no general recommendation for vaccination as outlined below. The live attenuated dengue vaccine Qdenga has been approved for travellers in the European Union (EU) in December 2022. The vaccine is based on an attenuated live virus of the dengue virus serotype 2 (DENV-2), being the genetic basis of the tetravalent vaccine. The vaccine was built to be effective against all four DENV serotypes. The primary immunization consists of 2 vaccine doses separated by 3 months and is licensed for use from the age of 4 years. The goal of the German vaccine recommendation is to prevent illness, severe clinical courses, and death due to a DENV infection. The German officials recommends vaccination against dengue with the vaccine Qdenga as a travel vaccination for individuals over the age of 4 years who have a history of a prior laboratory-confirmed dengue virus infection and who are travelling to a dengue-endemic region where they will have an increased risk of exposure (e.g., prolonged stay, current outbreak event). Prior to travel, a full vaccination series should be completed (i.e., two vaccine doses at a minimum interval of three months). The data for individuals who have not previously had a dengue virus infection (“dengue-naïve”) are very limited at present and there is risk of aggravated disease courses in vaccinated patients who had not suffered from a dengue virus infection before. Vaccination is therefore not generally recommended and should only be applied to patients with previous infections with dengue.

### Impact of vaccination programs – mycobacterium tuberculosis

In Germany, the BCG vaccination is not recommended for children or adults and is also not recommended as a travel vaccination. The German authorities are, thus, following the advice of the World Health Organization (WHO) not to carry out a general tuberculosis (TB) vaccination in countries with a tuberculosis infection risk of less than 0.1 percent. The effectiveness of the vaccine has probably diminished over the decades due to the repeated need to breed the original BCG bacteria. Despite these limitations, BCG vaccination remains one of the most important preventive measures against tuberculosis in high-risk areas for newborns and small children. The BCG vaccination is generally recommended for travelers only in specific, high-risk situations, such as children under 5 years, traveling to countries with a very high incidence of TB for an extended period. It is not recommended for general travel to most countries with a low TB risk. For those who should be vaccinated, the vaccine should be administered at least 3 months before travel.

### Impact of vaccination programs – rhabies

Also canine vaccination programs have decreased the incidence in several countries, rabies remains a significant neuroinfectious global health threat, characterized by an almost invariable fatality rate upon the onset of clinical symptoms [77]. The WHO estimates that still 60,000 patients die every year worldwide. However, the disease is entirely preventable through vaccination, timely Post-Exposure Prophylaxis (PEP) involving high-quality vaccines and, where indicated, rabies immunoglobulins [78]. Clinical data underscores the exceptional efficacy of modern cell-culture vaccines, which induce a robust neutralizing antibody response. When administered according to World Health Organization protocols, these vaccines provide near-total protection against viral entry into the central nervous system and recently, even single dose pre-exposure prophylaxis protocols have been proven effective (A. D. [35, 36]). For travelers to countries with a high burden of rhabies and a possible exposure to infected animals (e.g. because of outdoor activities), vaccination is recommended and should be taken into consideration.

## Conclusion

Prevention has a high impact on the incidence and course of many neuroinfectious diseases. Although successful vaccination programs against bacterial meningitis, tick borne encephalitis, and VZV infections have been rolled out, their effect would be much higher if sufficient vaccination rates would be achieved. From a neuroinfectious point of view, several issues need to be addressed to improve prevention: (i) adequate surveillance programs need to be established to create the basis for prophylactic interventions, (ii) promotion of available vaccination programs seems crucial to achieve adequate vaccination rates and (iii) further research on the prophylaxis and vaccine development of rare but life-threatening CNS infections needs to be supported.

The table provides exemplary data on the incidence of specific diseases before and after the implementation of vaccination programs in selected countries.

## Data Availability

Not applicable.

## Abbreviations

VZV: Varicella zoster virus
MIBE: Measles inclusion body encephalitis
SSPE: Subacute sclerosing panencephalitis
APME: Acute post-infectious measles encephalitis
PME: Primary measles encephalitis
CNS: Central nervous system
HiB: Haemophilus influenzae Type B
PCV: Pneumococcal conjugate vaccine
GBS: Group B Streptococcus
TBE: Tick-borne encephalitis
OspA: Outer surface protein A
GBS: Guillain barre syndrome
DENV: Dengue virus
TB: Tuberculosis
WHO: World health organization
